# *Fasciola hepatica* Extracellular Vesicles isolated from excretory-secretory products using a gravity flow method modulate dendritic cell phenotype and activity

**DOI:** 10.1371/journal.pntd.0008626

**Published:** 2020-09-08

**Authors:** Anna Murphy, Krystyna Cwiklinski, Richard Lalor, Barry O’Connell, Mark W. Robinson, Jared Gerlach, Lokesh Joshi, Michelle Kilcoyne, John P. Dalton, Sandra M. O’Neill

**Affiliations:** 1 Fundamental and Translational Immunology group, School of Biotechnology, Faculty of Science and Health, Dublin City University, Glasnevin, Dublin, Ireland; 2 School of Natural Sciences, Centre for One Health and Ryan Institute, National University of Ireland Galway, Galway, Ireland; 3 Nano Research, Dublin City University, Glasnevin, Dublin 9, Ireland; 4 Institute for Global Food Security, School of Biological Sciences, Medical Biology Centre (MBC), Queen’s University Belfast, Belfast, Northern Ireland, United Kingdom; 5 Glycoscience Group, Advanced Glycoscience Research Cluster, School of Natural Sciences, National University of Ireland, Galway, Ireland; 6 Carbohydrate Signalling Group, Discipline of Microbiology, National University of Ireland, Galway, Ireland; Wellcome Sanger Institute, UNITED KINGDOM

## Abstract

Parasite-released extracellular vesicles (EVs) deliver signals to the host immune system that are critical to maintaining the long-term relationship between parasite and host. In the present study, total EVs (FhEVs) released *in vitro* by adults of the helminth parasite *Fasciola hepatica* were isolated using a recently described gravity flow method that protects their structural integrity. The FhEVs molecular cargo was defined using proteomic analysis and their surface topology characterised by glycan microarrays. The proteomic analysis identified 618 proteins, 121 of which contained putative N-linked glycosylation sites while 132 proteins contained putative O-linked glycosylation sites. Glycan arrays revealed surface-exposed glycans with a high affinity for mannose-binding lectins indicating the predominance of oligo mannose-rich glycoproteins, as well as other glycans with a high affinity for complex-type N-glycans. When added to bone-marrow derived dendritic cells isolated FhEV induced a novel phenotype that was categorised by the secretion of low levels of TNF, enhanced expression of cell surface markers (CD80, CD86, CD40, OX40L, and SIGNR1) and elevation of intracellular markers (SOCS1 and SOCS3). When FhEV-stimulated BMDCs were introduced into OT-II mice by adoptive transfer, IL-2 secretion from skin draining lymph nodes and spleen cells was inhibited in response to both specific and non-specific antigen stimulation. Immunisation of mice with a suspension of FhEV did not elicit significant immune responses; however, in the presence of alum, FhEVs induced a mixed Th1/Th2 immune response with high antigen specific antibody titres. Thus, we have demonstrated that FhEVs induce a unique phentotype in DC capable of suppressing IL-2 secretion from T-cells. Our studies add to the growing immuno-proteomic database that will be an important source for the discovery of future parasite vaccines and immunotherapeutic biologicals.

## Author summary

Parasite-released extracellular vesicles (EVs) deliver signals to the host immune system that are critical to maintaining the long-term relationship between parasite and host. This study isolated total EVs (FhEVs) released *in vitro* by the adult stages of the parasitic worm *Fasciola hepatica* using a gravity flow method that protects the structural integrity of the vesicles. Proteomic analysis identified 618 proteins, 121 of which contained putative N-linked glycosylation sites while 132 proteins contained putative O-linked glycosylation sites while glycan arrays revealed surface-exposed glycans were predominantly oligo mannose-rich glycoproteins, and glycans with a high affinity for complex-type N-glycans. Since the EV molecular cargo can influence host immune cells, FhEVs were added to bone-marrow derived dendritic cells, inducing a novel cell phenotype that when adoptive transferred into OT-II mice inhibited IL-2 secretion from skin draining lymph nodes and spleen cells. Immunisation of mice with FhEV did not elicit significant immune responses; however, in the presence of alum, FhEVs induced a mixed Th1/Th2 immune response with high antigen specific antibody titres. This studied sheds like on the biological activity of FhEVs and added to the growing immuno-proteomic database that will be an important source for the discovery of future therapeutics.

## Introduction

Extracellular vesicles (EVs) are small membrane-bound vesicles secreted by multiple cell types as part of normal cellular bioprocesses [1–3]. EVs are important mediators of biological processes that act through an array of signalling components, including proteins, glycoproteins, lipids, and microRNAs [4–6]. They play a significant role in immune regulation [1,7], cell-cell communication [6], tissue repair [8], protection from injury, and blood clotting [9]. However, they are also implicated in pathological conditions by contributing to tumour proliferation, angiogenesis, metastasis, immune suppression, and therapeutic resistance [10–12]. A number of studies have proposed an important role for EVs in host-parasite interactions, particularly in modulating host immune responses [13,14].

EVs released from protozoa, such as Plasmodium [15,16], Trichomonas [17], and Leishmania [18,19] can influence the immune response in the host in order to enhance parasite survival in a variety of ways, such as mediating parasite-parasite and host-parasite communication, modulating parasite differentiation, and transferring virulence molecules to host cells [13–17]. In the case of worm parasites (helminths), isolation and characterisation of EVs from *Ascaris suum* [20], *Echinococcus granulosus* [21], *Ospisthorchis viverrini* [22], *Teladorsagia circumcincta* [23], *Heligmosomoides polygyrus* [18], *Schistosoma japonicum* [19], and *Fasciola hepatica* [24,25] suggest that these vesicles interact with host cells and contain molecules that exhibit immune regulatory properties [18,19,21,22,25]. EVs from *H*. *polygyrus* can suppress macrophage activation while *Schistosoma japonicum*-derived exosomes induce the activation of inflammatory M1-type macrophage activity [18,19]. *E*. *granulosus* secretes EVs that are internalised by dendritic cells enhancing CD86 and MHC II expression on the cell surface [21]. Recently, Roig *et al*. [26] showed that *F*. *hepatica* EVs modulate the activity of macrophages and DCs in addition to reducing the severity of DSS-induced colitis in mice suggesting that EVs contain potential biotherapeutic molecules. Many of these vesicles are targeted by antibodies isolated form infected animals [23,27] or animals vaccinated with EVs or EV-surface proteins [23,27] making them important vaccine candidates.

The parasitic worm *Fasciola hepatica* causes huge economic losses to global agriculture as one of the most common infections of livestock globally. Fascioliasis leads to weight loss, infertility, and poor growth rate estimated to cost the industry $3 billion annually [28]. *F*. *hepatica* is also a serious zoonotic disease that is estimated to affect 17 million people in under-developed rural communities, leaving infected people at risk of chronic infection and susceptible to secondary bystander microbial infections [24,29]. Resistance to triclabendazole, the long-standing gold standard chemotherapeutic treatment, means that the development of new chemotherapies [30], or better still, vaccines [24] is critical. Most past research has focused on the molecules associated with the surface tegument and secretome of *F*. *hepatica* as the main sources of vaccine candidates [31,32]. However, because of their aforementioned importance in host-parasite interaction, attention is now turning to EVs and their cargo for the discovery of novel therapeutic targets [25,33,34]. Indeed, a proteomic analysis of FhEVs by Cwiklinski *et al*. [34] identified several bioactive cargo molecules including peroxiredoxin, cathepsin B, cathepsin L1, and helminth defence molecule that exhibit immuno-modulatory properties such as the induction of M2 macrophages [35] and the suppression of inflammatory immune responses [36]. All of these molecules are considered targets at which new vaccines could be directed to prevent the parasite controlling the host immune responses [25,29,33,34] and, therefore, it is important to develop methods for the isolation and detailed molecular analysis of FhEVs, including their glycoproteins and glycans, and examine their immune-potentiating properties.

We exploited a gravity flow procedure, described by Muscante *et al*. [37], to isolate total EVs from the excretory-secretory (ES) products of adult *F*. *hepatica*. The advantage of this method is that vesicle structure and integrity remain intact ensuring that the biological activity of the vesicle remains preserved [38]. The method is particularly appropriate for the analysis of highly dilute samples and, therefore, does not require concentration and centrifugation steps or the addition of chemicals [39]. The first aim of this study was concerned with profiling the FhEVs composition using proteomic analysis, characterising their surface glycan topology using lectin arrays, and comparing these findings with FhEVs isolated by differential centrifugation. A second aim was to examine the immune responses of animals to FhEVs. When cultured with bone marrow-derived dendritic cells (BMDCs), the FhEVs induced a novel DC population capable of supressing T-cell responses *in vivo*. Furthermore, in the presence of adjuvant, FhEVs induced antigen-specific adaptive immune responses with high antibody titres. Our studies offer new methodologies for isolating intact EVs, show that these have immunodulatory properties and add to the rapidly growing immuno-proteomic database that can be interrogated by researchers for future parasite vaccines and immunotherapeutic biologicals.

## Materials and methods

### Animals and ethics

BALB/c and C57BL/6-Tg(TcraTcrb)425Cbn/Crl (OT II) mice aged 6–8 weeks were purchased from Charles River, UK, Ltd (Kent, UK) and kept under specific pathogen free conditions at the Dublin City University Bioresources unit. All mice were housed according to the Health Products Regulatory Authority guidelines and standard operating procedure approved by the institutional Animal Welfare Body were strictly adhered too. Ethical permission for the use of animals was approved by the Department of Health or Health Products Regulatory Authority (HPRA) and Dublin City University ethics committee (licence numbers B100/2833, DCUREC/2010/033). All procedures involving animals were only performed by licenced personnel.

### Preparation *F*. *hepatica* EVs by gravity flow

An adaption to the protocol described by Muscante *et al*. [37] that employs gravity flow to isolate EV populations was used in this study. Adult liver fluke were obtained from the liver of infected sheep at a local abattoir and washed with sterile PBS before culturing in RPMI medium (containing 0.1% glucose, 100 U penicillin and 100 mg/ml streptomycin) at a ratio of 1 worm / 2 ml at 37°C and 5% CO_2_. After 5 hours, culture media (excretory/secretory, ES, products) was collected and centrifuged at 300 x g for 10 mins and then 700 x g for 30 mins to eliminate large debris.

The supernatant (200 mls) was decanted into a sterile separating funnel attached to dialysis tubing with at molecular weight cut-off (MWCO) of 1000 kDa. The dialysis tubing was clamped at the bottom with a plastic clip and the set-up held in a class II biosafety cabinet. The hydrostatic pressure of the solution in the funnel forces the solution through the dialysis membrane. The solution was rinsed three times with 100 ml sterile PBS and allowed to concentrate to a volume of 10 ml. The solution was then transferred from the dialysis membrane into a sterile syringe and filtered through a 0.2 μm filter. This filtrate containing total FhEVs was aliquoted and stored at -80°C. FhEV protein concentration was estimated using a BCA commercial kit following treatment of the exosomes in RIPA, 10% Triton-X 100 in PBS and heating for 5 minutes at 100°C.

For comparison, FhEVs samples isolated by differential centrifugation (15K and 120K) were prepared as previously described [34]. Briefly, 50 ml parasite culture media was collected and centrifuged at low speed (first at 300 x g/10 min, and then at 700 x g/30 min) to remove debris. The supernatant was decanted and centrifuged at 15,000 x g for 45 min at 4°C to obtain large vesicles (15K vesicles) in a Sorvall RC6 plus centrifuge using fixed angled rotor SS-34 (k factor: 750; ThermoFisher Scientific). The supernatants was decanted, filtered using a 0.2 μm ultrafiltration membrane, and ultra-centrifuged at 120,000 x g for1 h at 4°C to recover smaller vesicles (120K vesicles) in a WX Ultra 90 centrifuge using a fixed angled rotor T647.5 (k factor at maximum speed: 114; ThermoFisher Scientific). These vesicles were washed with PBS and stored at -80°C. The final supernatant, excretory-secretory products FhES, was aliquoted and stored at -80°C. Endotoxin levels were tested for all antigens and were less than the lower limit of detection in this assay (<0.01 EU/ml).

### Transmission electron microscopy

The EV suspension was pipetted onto copper transmission electron microscopy (TEM) grids (coated with carbon and Formvar films), and left to rest for ~15 seconds. Using a Gatan Cryo-Plunge apparatus, the excess fluid was blotted off and the sample was flash frozen by plunging into liquid ethane kept at 77°K by liquid nitrogen. The sample was kept under liquid nitrogen until needed, whereupon it was transferred to Gatan Cryo Stage. To reduce the amount of ice on the sample, the cryo stage temperature was set to 173°K and the sample was left for 60 minutes under the vacuum of the SEM, ensuring sublimation of the ice. A Hitachi S-5500 Field Emission Transmission Electron Microscope was used to examine the samples. A number of imaging conditions were tried in transmission and reflection modes. One of the more successful settings was 1 kV, with a probe current of 10 μA using the secondary electron detector.

### Mass spectrometry analyses of EVs

Protein digestion and mass spectrometry analyses were performed by the Proteomics Platform of the CHU de Québec Research Center (Quebec, Qc, Canada). EV samples were washed using an Amicon Ultra 3 kDa column with 50 mM ammonium bicarbonate buffer before being dried by evaporation in a SpeedVac (ThermoFisher Scientific). Protein samples were solubilized in 50 mM ammonium bicarbonate and 1% sodium deoxycholate and then reduced with 0.2 mM DTT at 37°C for 30 min and alkylated with 0.9 mM iodoacetamide at 37°C for 20 min. Trypsin digestion of the protein samples was performed in solution using 0.1 μg sequencing grade trypsin (Promega) overnight at 37°C. The trypsin reaction was stopped by acidification using 3% acetonitrile, 1% TFA and 0.5% acetic acid. The digested peptides were purified by stage tip (C18), vacuum centrifuge dried and then re-suspended in 0.1% formic acid. The re-suspended peptide samples were separated by online reversed-phase (RP) nanoscale capillary liquid chromatography (nanoLC) using a Dionex UltiMate 3000 nanoRSLC chromatography system (Thermo Fisher Scientific / Dionex Softron GmbH, Germering, Germany). Electrospray mass spectrometry (ESI MS/MS) analysis was performed on a Orbitrap Fusion mass spectrometer (Thermo Fisher Scientific, San Jose, CA,USA) utilising the Orbitrap Fusion Tune Application 2.0 and fitted with a nanoelectrospray ion source.

MS/MS peak lists (MGF files) were generated using Thermo Proteome Discoverer software (Thermo Fisher Scientific Inc., version 2.2.0) and analysed using Mascot (Matrix Science, London, UK; version 2.5.1), set up to search against a database comprised of gene models identified from the *F*. *hepatica* draft genome (version 1.0, 101,780 entries; PRJEB6687; [27]), assuming digestion with trypsin with two missed cleavages permitted. Fragment and parent ion mass tolerance were set at 0.60 Da and 10.0 PPM, respectively. Carbamidomethyl of cysteine was set as a fixed modification and the deamidation of asparagine and glutamine, and oxidation of methionine specified as variable modifications.

Scaffold (version 4.8.4, Proteome Software Inc., Portland, OR) was used to validate MS/MS based peptide and protein identifications. Peptide identifications were accepted if they could be established at greater than 95% probability to achieve an FDR less than 1.0% by the Scaffold Local FDR algorithm. Protein identifications were accepted if they could be established at greater than 95.0% probability to achieve an FDR less than 1.0% and contained at least 2 identified peptides. Protein probabilities were assigned by the Protein Prophet algorithm [40]. Proteins that contained similar peptides and could not be differentiated based on MS/MS analysis alone were grouped to satisfy the principles of parsimony. Putative annotation of the *F*. *hepatica* gene models was assigned using *in silico* tools; Uniprot, Gene Ontology (GO) and InterproScan [34]. The identified proteins in this study were categorised according to their GO classification. Prediction of signal peptide sequence and glycosylation sites within proteins putatively associated with the FhEV surface was carried out using SignalP 4.1 Server (http://www.cbs.dtu.dk/services/SignalP-4.1/), NetNGlyc 1.0 Server (http://www.cbs.dtu.dk/services/NetNGlyc/) and NetOGlyc 4.0 Server (http://www.cbs.dtu.dk/services/NetOGlyc/), respectively. Protein enrichment analyses were carried out using the STRING database based on *Schistosoma mansoni* proteins [41].

### Analysis of the FhEVs surface glycans topology by lectin microarray

FhEVs isolated by centrifugation or gravity flow were fluorescently labelled with the lipophillic dye PKH26 (Sigma Aldrich, Dublin) as previously reported [42] with the current protocol omitting BSA as the termination of the labelling sequence. All labelling was carried out at 23°C in the dark. Following labelling, excess dye was removed from each portion of FhEVs by centrifugal filtration in a 500 μl, 100 kDa MWCO spin device (Amicon, EMD-Millipore, Cork, Ireland). Each MWCO device was pre-washed with PBS containing 0.1% BSA and the entire final volume of the FhEV labelling mixture added. An additional 2 ml PBS was added prior to centrifugation for 20 minutes, 8000 x g, at room temperature. Finally, an additional 1 ml of PBS was added to the spin device prior to the final centrifugation at 8000 x g in which the final volume was reduced to approximately 50 μl. Retentate containing labelled FhEVs was removed by pipetting. The bottom of each spin device was rinsed with an additional 20 μl of PBS which was added to the recovered FhEV volume. Labelled FhEVs were used for lectin microarray analysis immediately. Lectin microarrays (v2.4.0) with an extensive lectin panel were generated as previously described [42]. FhEVs were diluted to a final working concentration of 8.6 μm ml^-1^ with Tris-buffered saline supplemented with Ca^2+^ and Mg^2+^ ions (TBS; 20 mM Tris–HCl,100 mM NaCl, 1 mM CaCl_2_, 1 mM MgCl_2_) pH 7.2 with 0.05% Tween-20 (TBS-T). PKH26 FhEV was applied to each well of the gasket and incubated for 1 h at 23°C (4 rpm); slide washes were carried out as previously described [43]. Slides were dried by centrifugation and imaged immediately in an Agilent G2505 microarray scanner (Agilent Technologies) using the Cy3 channel (532 nm excitation, 80% PMT, 5 μm resolution).

Lectin microarray data extraction was performed as previously described [42]. In brief, raw intensity values were extracted from the image files using GenePix Pro v6.1.0.4 (Molecular Devices) and a proprietary address file to identify printed lectin positions using adaptive diameter (70–100%) circular alignment based on 230 μm features and exported as text to Excel (version 2013, Microsoft). Local background-corrected median feature intensity data (F532median-B532) was selected and the median of six replicate spots per subarray was handled as a single data point for graphical and statistical analysis (n = 4). All data was subjected to total intensity median correction normalization prior to analysis in Excel. Binding data was presented as the mean intensity with standard deviation of four experimental replicates (24 data points in total). A heat map demonstrating relative normalized intensity for each replicate was prepared with Hierarchical Clustering Explorer v3.5 (http://www.cs.umd.edu/hcil/multi-cluster/).

### Generation and activation of BMDCs

Bone marrow derived cells (BMDCs) from BALB/c and OT-II mice were differentiated as previously described [44]. Briefly, bone marrow was harvested from the tibia and fibula of a mouse by flushing with PBS. Cells were washed and re-suspended in complete RPMI (RPMI supplemented with 10% FCS, 1% L-glutamine, 1% penicillin/streptomycin) and 20 ng/ml GM-CSF. The media was replenished on days 3, 6 and 8, and the cells were harvested for experimental use on day 10. DC purity was assessed by CD11c expression by flow cytometry, and only used if purity was greater than 95%. BMDC cell number and viability was determined by trypan blue staining. Viable BMDCs were then re-suspended to a final concentration 1 x 10^6^/ml in complete RPMI containing 2 ng/ml GM-CSF and treated with FhES (10 μg/ml) or FhEVs (10 μg/ml) for 2.5 hours prior to stimulating with and without LPS (100 ng/ml). A dose of FhEVs at 10 μg/ml was selected following a dose response study measuring TNF-α. After 18 hours supernatants were removed and analysed for cytokine secretion using commercial ELISA kits for TNF-α, IL-12 and IL-10, and cells were removed for cell surface marker analysis using flow cytometry.

For qPCR analysis BMDCs were stimulated with PBS or FhEVs for 30 minutes and 2.5 hours for SOCS1 and SOCS3 expression respectively. For cytokine blocking experiments, BMDCs were incubated with anti-TLR2 (20 μg/ml) and anti-TLR4 (20 μg/ml) for 30 minutes prior to FhEVs stimulation for 18 hours.

### Adoptive transfer of FhEVs stimulated BMDCs into OT-II mice

To determine if FhEVs-stimulated BMDCs can influence T-cell priming *in vivo*, BMDCs from OT-II mice were isolated and stimulated with PBS or FhEVs (10 μg/ml) in the presence of ovalbumin (OVA) peptide (100 ng/ml) (Sigma Aldrich, Dublin, Ireland). After 24 hours cells were washed three times in sterile PBS and 3 x 10^5^ BMDCs were delivered over the sternum of naïve OT-II mice by subcutaneous injection. After 7 days mice were sacrificed by cervical dislocation and skin draining lymph nodes (sdLN) and spleens were removed and a single cell suspension, 1 x 10^6^/ml sdLN and 5 x 10^6^/ml spleen, was prepared, plated and re-stimulated with PBS, OVA peptide (500 ng/ml) or PMA (20 ng/ml) (Sigma Aldrich, Dublin, Ireland) and anti-CD3 (1 μg/ml). After 72 hours supernatants were removed for measurement of cytokines IL-2, IFN-γ and IL-13 by commercial ELISA kits.

### Flow cytometry

Cells were harvested after stimulation, washed twice in FACS buffer (PBS, 2% FCS, 1 mM EDTA) and then incubated with the following mouse antibodies: CD80 (PE), CD86 (FITC), CD40 (PE), OX40L (PE), SIGNR1 (APC), MR (APC), Dectin-1 (FITC) and ICAM-1 (PE) or the relative isotype control (eBioscienes, Hatfield, UK) for 30 minutes at 4°C in the dark. The cells were then washed twice with FACS buffer and analysed on a BD FACS Aria. The data was analysed using Flow Jo software (Treestar, Ashland, USA). In all experiments unstained and single stained controls were used for gating and compensation.

### Q-polymerase chain reaction

RNA from control and FhEVs stimulated BMDCs was isolated using a high-pure RNA isolation kit according to manufacturer’s guidelines (Roche, UK). The quality and quantity of RNA was assessed using Nanodrop (ThermoFisher Scientific UK). RNA was reverse transcribed to cDNA using the Transcriptor first strand cDNA synthesis kit (Roche Diagnostics, UK) with random hexamer primers. Primer probes were used to detect the expression of specific genes listed in Table 1. Two housekeeping genes were used as internal standards, GAPDH (NM_008084.2) and β-actin (NM_007393.1). Experiments were carried out in triplicate with each reaction containing 50 ng of cDNA, 1 μM of primer probe and 10 μl of FastStart Essential DNA Probes master mix (Roche Diagnostics, UK) containing a 6-carboxyfluorescein labelled enzyme, dNTP mix and MgCl2. Reaction volumes were brought up to a final reaction volume of 20 μl with PCR grade H2O (Roche Diagnostics, UK). Gene expression was analysed using a Light Cycler 96 (Roche, UK), using the following cycling conditions; an initial denaturation step at 95°C for 10 s, followed by 40 cycles at 95°C for 15 s and 60°C for 60 s. Pfaffl’s methods were used to determine relative gene expression [45] whereby the comparative cycle threshold (Ct) values of the samples of interest are compared with a control and normalised to the housekeeping genes.

**Table 1:** Forward and reverse primer sequences used for qPCR analysis.

Gene Name	Forward	Reverse
GAPDH	AGCTTGTCATCAACGGGAAG	TTTGATGTTAGTGGGGTCTCG
Β-actin	GGATGCAGAAGGAGATTACTGC	CCACCGATCCACACAGAGTA
SOCS1	GAGTGGTTGTGGAGGGTGAG	TGAGAGGTGGGATGAGGTC
SOCS3	ATTTCGCTTCGGGACTAGC	AACTTGCTGTGGGTGACCAT

### Immunisation protocol

For immunisation studies 6–8 BALB/c mice were injected intraperitoneally on day 0, 14 or 28 with PBS, FhEVs (10 μg per mouse) or FhES (10 μg per mouse) with or without Alum (100 μg per mouse). Mice were sacrificed by cervical dislocation two weeks after final immunisation and blood samples obtained post mortem. Blood was left to settle in 1.5 ml tubes at 4°C for 2 hours and samples centrifuged at 300 x g for 5 minutes to collect sera which was stored at -80°C until required.

To determine specific antibody responses to FhEVs, serum levels of total IgG, IgG1 or IgG2a antibodies were detected by indirect ELISA as previously described [46]. In brief, 96-well plates were coated overnight at 4°C with 10 μg/plate (1 μg/ml) of FhEVs, FhES, Peroxiredoxin (FhPRX) or stefin 1 (FhStf-1) (FhPRX and FhStf-1 are recombinantly made proteins, 2 of the top 30 proteins identified in FhEVs by abundance (Table 3). Plates were washed three times in 0.1% PBS-T and then blocked with 5% skimmed milk in PBS-T for 1 hour at 37°C. The wash step was repeated prior to samples being serially diluted (1:200 to 1:12800) in PBS-T in triplicate. Plates were incubated for 2 hours at room temperature and then the wash step was repeated prior to adding 100 μl of horseradish peroxidase conjugated goat anti-mouse IgG, IgG1 or IgG2a antibodies for 1 hour at room temperature. After incubation, plates were washed three times and incubated with 100 μl substrate solution. The reaction was stopped with 50 μl 2 N sulphuric acid and the absorbance read at 450 nm on a TECAN GeniosMicroplate Reader (Tecan, Mannedorf, Switzerland).

To measure cellular immune responses, the spleens of immunised mice were removed and a single cell suspension, 5 x 10^6^/ml splenocytes, was prepared, plated and re-stimulated with PBS, FhEVs (10 μg/ml), FhES (10 μg/ml) (or PMA (20 ng/ml) (Sigma Aldrich, Dublin, Ireland) and anti-CD3 (1 μg/ml). After 72 hours supernatants were removed for measurement of cytokines IFNγ, IL-2, IL-5, Il-10 or IL-17 by commercial ELISA kits.

### Data analysis

All data were analysed for normality prior to statistical testing by Prism 6.0 (GraphPad Software Inc, La Jolla, CA, USA) software. Where multiple group comparisons were made, data were analysed using two-way ANOVA using Tukey’s multiple comparison test. For comparisons between two groups, the Student’s *t* test was used. In all tests, *p* < 0.05 was deemed significant.

## Results

### Characterisation of *F*. *hepatica* EV populations isolated by gravity flow

**Fig 1:**
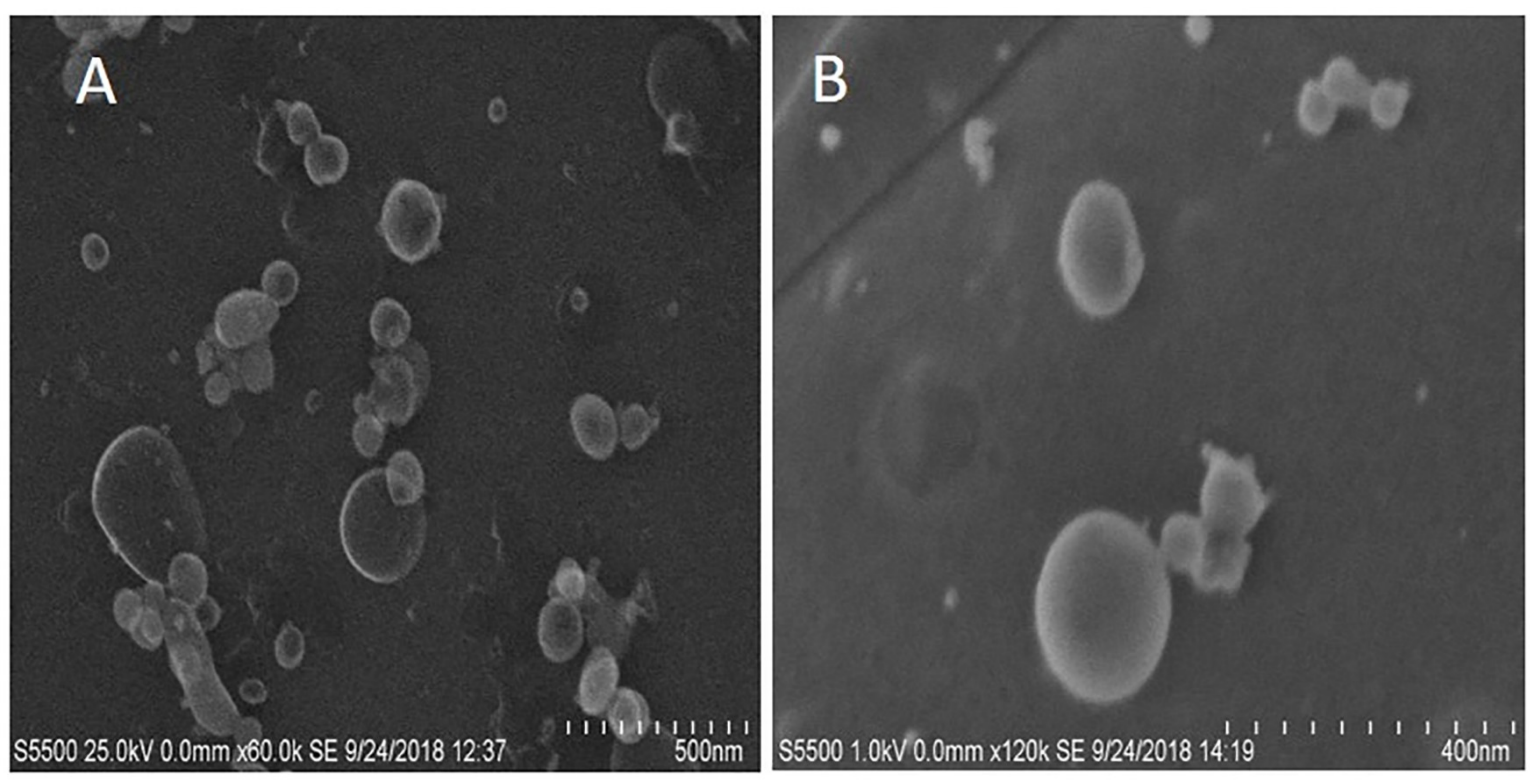
Transmission electron microscopy of FhEVs. An image of FhEVs as seen by transmission electron microscopy (TEM) at different magnifications. A and B are two different sample preparations.

Total FhEVs produced by adult *F*. *hepatica* in culture were captured using a protocol adapted from Muscante *et al*. [37] that employs simple gravity flow to non-disruptively concentrate EV populations inside a dialysis bag with a molecular weight cut-off of 1000 kDa. The size of the EVs in this preparation were determined using TEM, and shown to range from 30 to 200 nm in diameter indicating the varied nature of the EVs released by this parasite *in vitro* (Fig 1).

A proteomics approach was taken to characterise the protein make-up of the FhEVs; 618 proteins were identified with at least two unique peptides across both technical replicates (S1 Table). Consistent with the source of the preparation, gene ontology (GO) analysis identified proteins associated with GO terms related to extracellular vesicles, including vesicle mediated transport (GO:0016192), vesicle fusion (GO:0006906), extracellular exosome (GO:0070062), exocytosis (GO:0000145; GO:0006887), endocytosis (GO:0006897) and the ESCRT I complex (GO:000813) (S2 Table). Similarly, KEGG pathway analysis revealed that the endocytosis pathway was significantly enriched within the proteins identified from the gravity-flow FhEVs (Table 2).

**Table 2:** KEGG pathways significantly enriched within total EV proteome (618).

KEGG pathway	Description	Observed	FDR*
Total protein			
**4144**	Endocytosis	26	3.97E-19
**1120**	Microbial metabolism in diverse environments	18	1.68E-09
**1200**	Carbon metabolism	17	3.45E-09
**4145**	Phagosome	13	4.09E-08
**10**	Glycolysis / Gluconeogenesis	10	6.68E-07
**1100**	Metabolic pathways	44	6.68E-07
**590**	Arachidonic acid metabolism	6	5.39E-06
**480**	Glutathione metabolism	7	6.66E-06
**30**	Glutathione metabolism	7	1.84E-05
**1230**	Glutathione metabolism	8	7.09E-04
**4142**	Lysosome	9	8.27E-04
**51**	Fructose and mannose metabolism	5	0.00154
**620**	Pyruvate metabolism	6	0.00165
**270**	Cysteine and methionine metabolism	4	0.0213
**2010**	ABC transporters	3	0.0249
**630**	Glyoxylate and dicarboxylate metabolism	3	0.0471

*FDR–False Discovery Rate

In addition to the EV-associated/specific proteins, we also identified soluble proteins associated with the cargo of the FhEVs including several peptidases (cathepsin L and B, leucine aminopeptidases), cysteine peptidase inhibitors (stefin 1, multidomain cystatin, FhKT1 kunitz inhibitor), and serine peptidase inhibitors (serpins). Furthermore, major redox-based antioxidant enzymes were also identified including four classes of glutathione S-transferases (mu, sigma, omega, and zeta), fatty acid binding proteins, superoxide dismutase and the complete thioredoxin reductase-thioredoxin-peroxiredoxin cascade (S1 Table).

Analysis of the FhEV profile by protein abundance (emPAI) revealed several key molecules of interest were found to be particularly abundant components; therefore, the top 25 proteins, represented >50% of the total protein recovered from the gravity-flow FhEVs. Thioredoxin was found to be the most abundant protein within the EVs (Table 3), alone representing approximately 14% of the total protein in the FhEVs. In addition, leucine aminopeptidases, stefin-1, and a heat shock protein (HSP-70) were found amongst the most abundantly expressed cargo proteins. Major structural proteins universal stress proteins (USPs), annexins and syntenin-1 were also identified within the top 25 proteins expressed, as well as proteins associated with the FhEV surface [25,34] such as ubiquitin and stefin 1. Lastly, a number of uncharacterised proteins were found that may warrant further investigation to determine their role in FhEV uptake.

**Table 3:** The 25 most abundant proteins identified within the EV protein samples, based on protein abundance calculated by emPAI.

ID	Description	Unique peptide*	emPAI value*
**BN1106_S4026B000080**	Thioredoxin^	12	267.45
**BN1106_S1026B000543**	Universal stress protein	12	126.04
**BN1106_S6576B000103**	Ubiquitin	5.5	114.46
**BN1106_S390B000196**	Syntenin-1	14.5	68.26
**BN1106_S1806B000293**	Uncharacterised^#^	4	66.22
**BN1106_S617B000566**	Leucine aminopeptidase^	30	55.28
**BN1106_S586B000374**	Natterin/DM9 domain containing protein	11	46.14
**BN1106_S309B000234**	HSP 70^	38.5	29.52
**BN1106_S819B000364**	Annexin^	27	29.07
**BN1106_S1300B000145**	Radixin	38.5	25.19
**BN1106_S6840B000044**	Uncharacterised^	2	24.00
**BN1106_S4131B000138**	GLIPR1-like protein 1	10	23.37
**BN1106_S1819B000120**	Serine/threonine-protein kinase	19.5	22.00
**BN1106_S6006B000040**	Cubulin	6	21.49
**BN1106_S4651B000094**	Cystatin-1	6.5	20.21
**BN1106_S4672B000098**	Rho GDP-dissociation inhibitor	10	20.17
**BN1106_S1172B000096**	RAB 27A	8	17.77
**BN1106_S1444B000095**	Dynein light chain^#^	6.5	17.44
**BN1106_S2655B000264**	Charged multivesicular body protein	13	15.39
**BN1106_S175B000200**	Hexokinase^	28	15.16
**BN1106_S551B000321**	Uncharacterised	17.5	14.91
**BN1106_S617B000567**	Leucine aminopeptidase	8.5	14.13
**BN1106_S3747B000112**	IST1 homolog	15	12.63
**BN1106_S1871B000313**	Programmed cell death 6 interacting protein (ALIX)	47	12.47
**BN1106_S440B000223**	Uncharacterised	11	12.45
**BN1106_S1679B000169**	Universal stress protein	9.5	11.90

*Representing mean values between technical replicates.

^#^ Representing proteins unique to the gravity flow method.

^ Proteins abundantly expressed in EVs isolated by differential centrifugation [27–28].

### Comparative analysis *F*. *hepatica* EV populations isolated by gravity flow versus differential centrifugation

**Fig 2:**
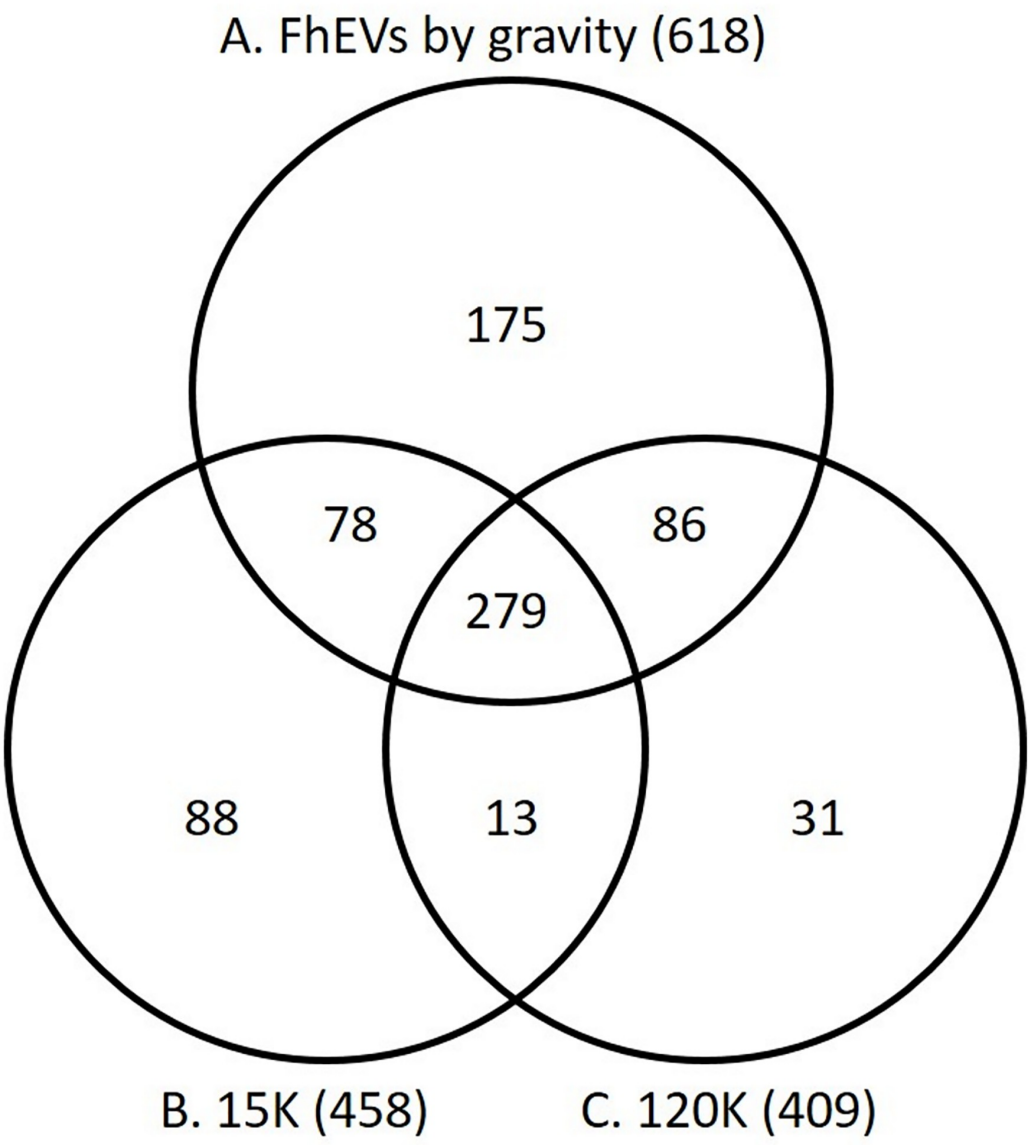
Expression of proteins in FhEVs isolated with gravity compared to FhEVs isolated using differential centrifugation. Venn diagram comparing the proteins identified in FhEVs isolated with gravity (A) compared to FhEVs isolated using centrifugation at at 15,000 x g (B; 15K) or 120,000 x g (C; 120K), based on proteins identified by unique peptide count. The numbers in brackets represent the total number of proteins from each dataset.

We compared the proteomic profile of the gravity flow FhEVs with that of FhEVs isolated from adult *F*. *hepatica* products by differential centrifugation methods we previously published [25,34]. Using the centrifugation methodology, FhEVs can be subdivided into two sub-populations: (a) large EVs that are found in the pellet at 15,000 x g (15K), and (b) smaller exosome-like EVs that were recovered from the supernatant by ultra-centrifugation at 120,000 x g (120K). Comparative analysis of the complete datasets revealed that 72% of our 618 proteins were identified in the 15K and 120K EVs isolated by differential centrifugation (Fig 2; S1 Table). Of the top 25 most abundant proteins identified in the gravity-flow FhEVs, 14 proteins were also amongst the most abundant proteins of the 15K and 120K EVs, including the six proteins mentioned above, namely, thioredoxin, leucine aminopeptidase, HSP-70, annexin, hexokinase, and an uncharacterised protein (Table 2). This indicated that the EVs isolated by gravity-flow contained the vesicles typically recovered by differential centrifugation.

A total of 175 proteins were found to be only present within the FhEVs isolated by gravity flow. Conversely, a total of 132 proteins were identified in the 15K/120K FhEV preparations but not in the gravity-flow FhEVs [34]. However, functional annotation of these sets revealed that they were not novel proteins, but isoforms of proteins already identified within FhEV populations, reflective of the large number of expanded gene families within the *F*. *hepatica* genome [34]. Based on protein concentration (emPAI), the proteins exclusive to the gravity-flow FhEVs only contributed 11% of the total protein isolated, represented mainly by two proteins, an uncharacterised protein (3.6%, BN1106_s1806B000293) and a dynein light chain protein (0.94%, BN1106_s1444B000095). Similarly, the additional 132 proteins identified in the differential centrifugation preparations represented a small proportion of the total protein, 12.9% and 2.8% for the 15K and 120 FhEVs, respectively. This analysis demonstrates that the FhEV isolated by gravity flow essentially consist of the same protein populations as the combined 15K and 120K FhEVs.

de la Torre-Escudero *et al*. [25] characterised the surface-exposed proteins of the 15K and 120K FhEVs and suggested that these may be involved in vesicle docking and internalisation into host cells. A comparative analysis showed that 183 proteins from the total 618 gravity-flow FhEVs proteins matched with proteins exposed to the vesicle surface (S3 Table). Also consistent with studies by de la Torre-Escudero *et al*. [25], a high proportion of these proteins were found to be putatively glycosylated, and could play a role in how these EVs are internalised by host cells; 121 proteins contained putative N-linked glycosylation sites while 132 proteins contained putative O-linked glycosylation sites (S3 Table). *In silico* analysis revealed that, only 17 of these proteins were found to contain a signal peptide. This result is consistent with previous analysis of *F*. *hepatica* EVs [34], and suggests that extracellular vesicles are an important alternative method of secretion for key immunomodulatory molecules lacking a secretory signal.

### The surface glycan topology of FhEVs is similar to that of both 15k and 120k vesicles

**Fig 3:**
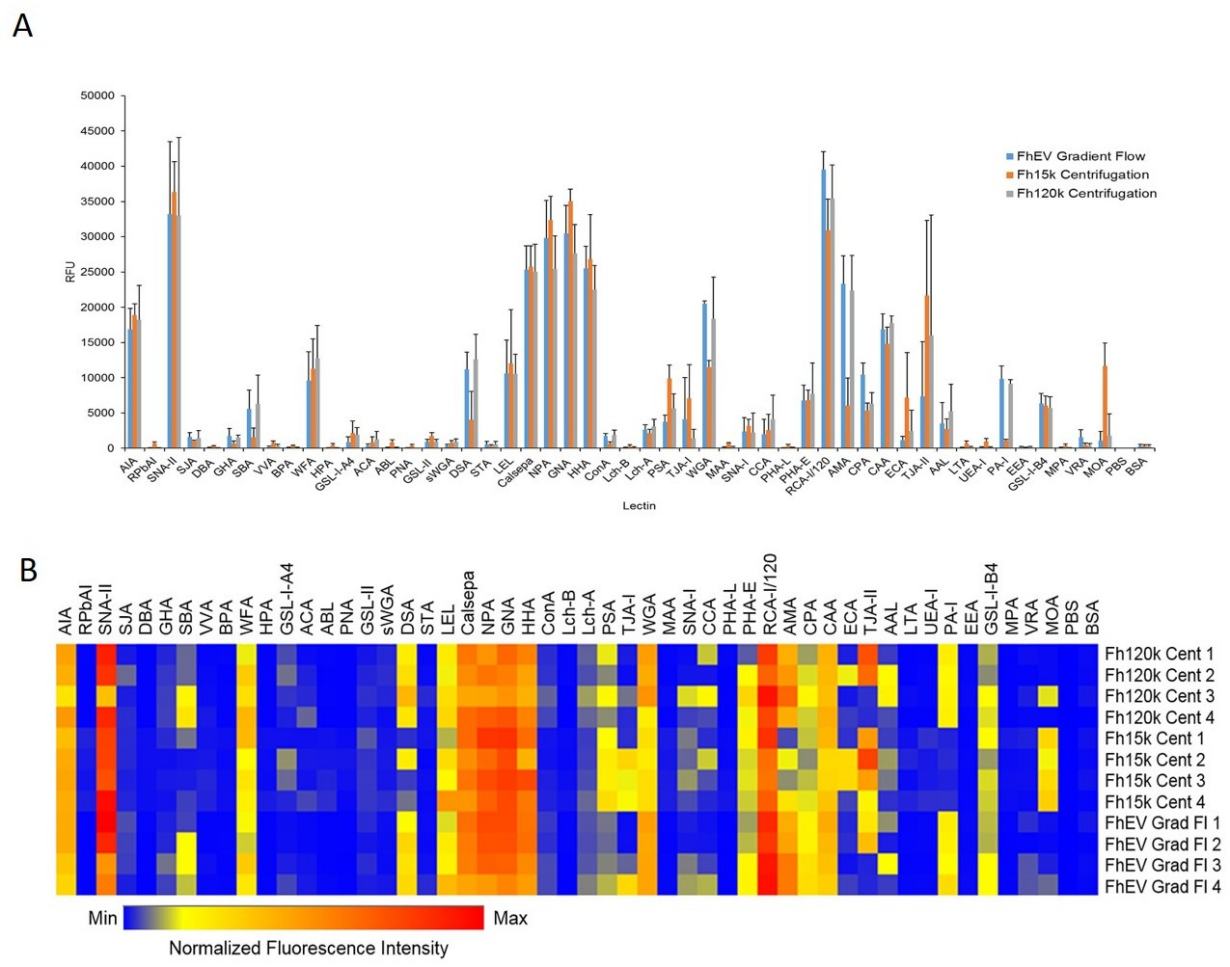
Lectin microarray of FhEVs isolated with gravity compared to FhEVs isolated using differential centrifugation. Fluorescently labelled FhEVs isolated by ultracentrifugation (Fh120k), high speed centrifugation (Fh15k) and gradient flow (FhEV) methods were incubated with a proprietary lectin microarray (1 hour, 23°C, 4 rpm) in TBS-T supplemented with 1mM Ca^2+^ and Mg^2+^. Slides were scanned spectrophotometrically using the Cy3 channel. Binding data is presented as **(A)** a bar chart, illustrating the mean intensity with standard deviation of four experimental replicates (24 data points in total) and **(B)** as a heat map, representing normalized individual sample intensity for each replicate.

We fluorescently labelled FhEVs isolated by gravity-flow and centrifugation with PKH26 and examined the surface glycan topology of the EVs by probing lectin microarray containing a comprehensive panel of lectins primarily derived from plant sources (S4 Table). Some differences in intensity were observed for FhEVs isolated by the different methods; however, in general, all three sets of FhEVs exhibited a high affinity for mannose-binding lectins (NPA, GNA, HHA, Calsepa, LEL, PSA), confirming the predominance of oligo mannose-rich glycoproteins and a high affinity for complex-type N-glycans (TJAII, ECA, CAA, AMA, and RCA1) (Fig 3A & 3B). FhEVs also bound to galactose (AIA and SNAII), GalNAc and GlcNAc binding lectins and to terminal α-linked galactose-binding lectins (most intensely GSL-I-B4 and MOA). In this study, SBA, PA-I, and MOA were slightly elevated in the 120K vesicles which differed from data presented by de la Torre-Escudero et al. (2019) [25]. However, the signals were of a relatively low intensity compared to other lectins that exhibited much stronger signals. Furthermore, there was a significant degree of variability between these samples (Fig 3A & 3B).

### FhEVs induce a distinct DC phenotype

Dendritic cells (DCs) are a heterogeneous population of prominent antigen presenting phagocytes that display an abundant range of recognition receptors enabling them to sense and respond to array of stimuli. Previous studies have shown that *in vivo F*. *hepatica* infection [47] or *in vitro* exposure to secretome [36,48] and tegument protein fractions [36,49] exert suppressive effects on DCs. We investigated immunomodulatory/immunostimulatory effects of total FhEVs isolated by gravity flow on BMDCs activation, maturation and function by measuring the expression of cytokines, cell surface markers, and suppressor of cytokine signalling 1 (SOCS1) & SOCS3 gene expression.

**Fig 4:**
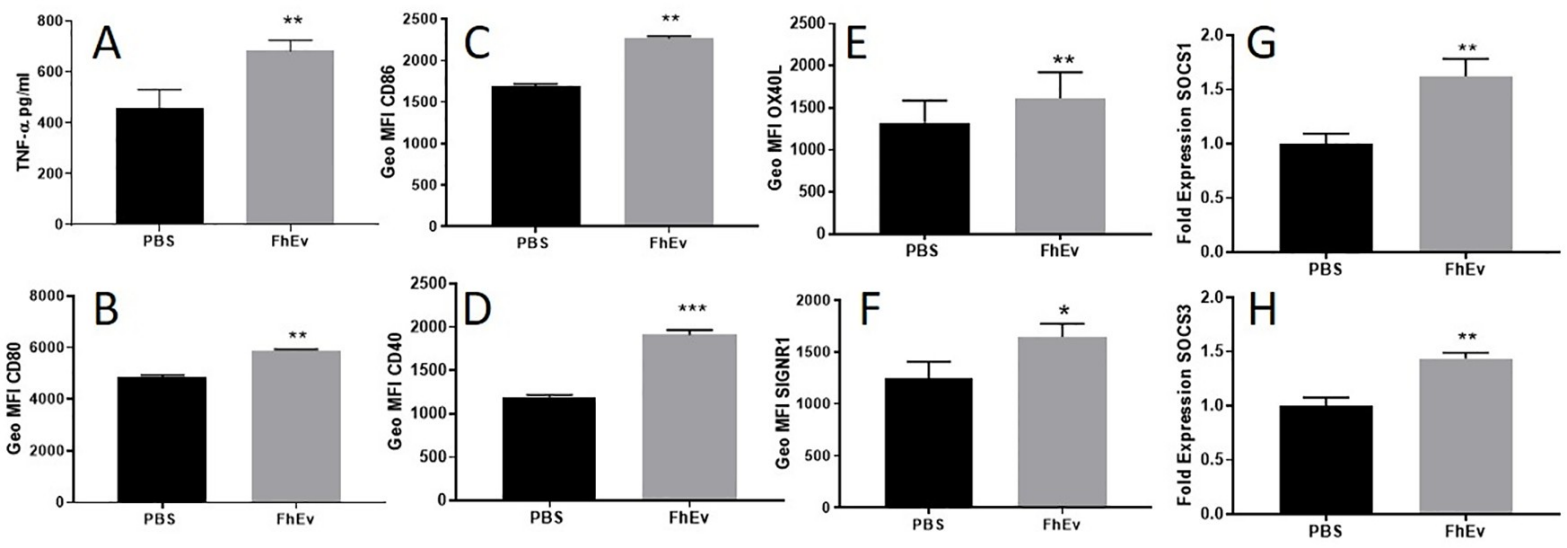
FhEVs induce a distinct BMDC phenotype. BMDCs were incubated with FhEVs for 18 hours and supernatant was removed to measure **(A)** TNF-α by commercial ELISA. Cell surface expression of **(B)** CD80, **(C)** CD86, **(D)** CD40, **(E)** OX40L and **(F)** SIGNR1 on FhEVs stimulated dendritic cells after 18 hours and **(G)** SOCS3 and **(H)** SOCS1 expression by RT-PCR. Data shown is a representative of three independent experiments presented as the mean ± SD of three replicate samples, *p<0.05, **p<0.01, *** p<0.0001. Student t-test was used for differences between means of control and treated (A-F) and between fold change between control and treated (G-H).

In response to an 18 hour incubation with FhEVs, BMDCs secreted significant levels of TNF-α (p<0.01, Fig 4A) but not IL-10 or IL-12p70 compared to PBS control (S1A–S1C Fig). By comparison, FhES did not induce any of the cytokines measured (TNFα, IL-10, and IL-12p70) compared to PBS control. To determine if the upregulation of TNF-α induced by FhEVs was TLR dependant, these experiments were repeated using anti-TLR4 and anti-TLR2 blocking antibodies, however no significant differences in TNF-α production was observed (S1D Fig).

FhEV-stimulated BMDCs also exhibited significant increased expression of several co-stimulatory markers for T-cell communication, including CD80 (p<0.01), CD86 (p<0.01), CD40 (p<0.001), SIGNR1 (p<0.05), and OX40L (p<0.01) compared to the PBS control (Fig 4B–4F). However, FhEVs did not induce an increase in the expression of Dectin-1, Mannose receptor (MR) or ICAM1 on BMDCs (S1E and S1F Fig).

Although some studies have shown that helminth-derived antigens suppress cytokine production in LPS stimulated DCs [35,36], we found that FhEVs and FhES, did not suppress cytokine production of TNF-α, IL-12p70 or IL-10, in LPS-stimulated BMDCs compared to LPS-treated BMDC controls (S1A–S1C Fig).

We examined the expression of SOCS1 and SOCS3 in FhEV treated BMDCs because we previously found that these regulators of cytokine expression are associated with *F*. *hepatica* infection [50]. DCs stimulated with FhEVs showed significant expression of SOCS3 (p<0.01; Fig 4G) and SOCS1 (p<0.01; Fig 4H) compared to the un-stimulated control.

### FhEV-stimulated DCs modulate cytokine secretion in CD4^+^ T-cells *ex vivo*

**Fig 5:**
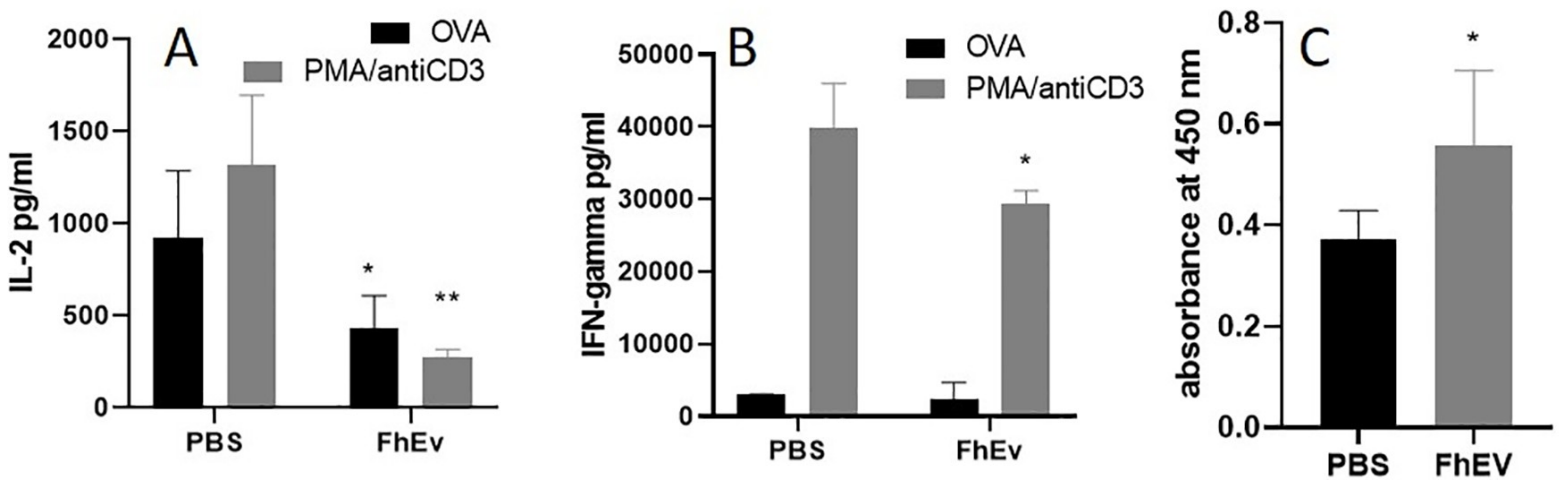
Adaptive immune responses to FhEVs. BMDCs treated with OVA peptide were also adoptively transferred over the sternum of OT-II mice. After 7 days, sdLN were removed for re-stimulation with OVA peptide or PMA (20ng/ml) and anti-CD3 (1μg/ml) for 72 hours and **(A)** IFN-γ and **(B)** IL-2 measured by commercial ELISA. 6 Mice were injected with PBS or FhEVs on day 0 and 14. After 2 weeks’ serum was isolated to measure Total IgG specific FhEV antibody responses (C). Data shown is the mean ± SD of three replicate samples from 8 mice, *p<0.05, **p<0.01, *** p<0.001. For multiple comparisons, data was analysed by two-way ANOVA using Tukey’s multiple comparison test (A, B). Otherwise, student t-test was used for differences between means of control and treated (C).

Given the observed modulatory effects of FhEVs on BMDC co-stimulatory molecule expression, we examined the capacity of FhEVs-treated DCs to prime T-cell responses. DCs were stimulated overnight with PBS or FhEVs in the presence of OVA peptide and then adoptively transferred into naïve OT-II mice. After 7 days, cells in skin-draining lymph nodes (sdLN) were isolated and re-stimulated with PBS, OVA peptide or PMA and anti-CD3 for 72 hours and cytokine production measured. We found that IL-2 secretion was significantly reduced (p<0.05) in FhEV-stimulated DC recipient mice in response to re-stimulation with OVA peptide and PMA and anti-CD3 (p<0.01) showing that FhEVs can modulate IL-2 secretion in response to both specific and non-specific antigen stimulation (Fig 5A). sdLN did not show any change in IFN-γ levels compared to OVA only treated DC recipient mice; however, a significant reduction in IFN-γ (p<0.05) levels was observed in PMA and anti-CD3 stimulated cells indicating a more general immuno-suppressive effect (Fig 5B). No significant differences in antigen-specific responses were observed for IL-10 and IL-13 (S1H and S1I Fig).

### BALB/c mice immunised with FhEVs formulated with alum induce mixed Th1/Th2 adaptive immune response

**Fig 6:**
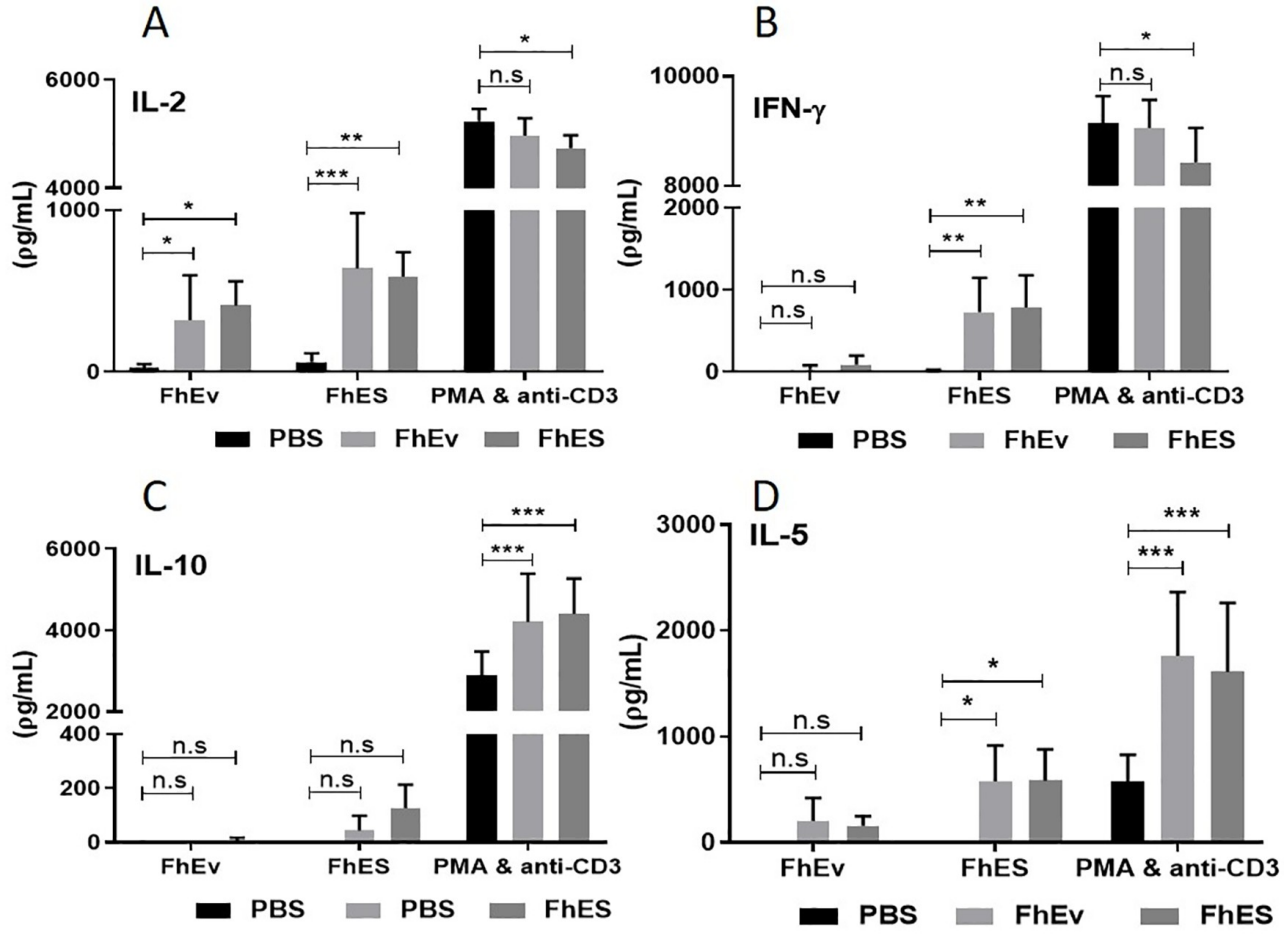
Mice immunised with FhEVs formulated in Alum display mixed Th1/Th2 immune response. 5–6 Mice were injected with PBS, FhEVs or FhES (all in alum) on day 0, 14 and 28. After 2 weeks’ spleens were removed for re-stimulation with PBS, FhEVs, FhES or PMA (20ng/ml) and anti-CD3 (1μg/ml) for 72 hours and IL-2 (A), IFN-γ (B), IL-10 (C) and IL-5 (D) measured by commercial ELISA. Data shown is presented as the mean ± SD of triplicate samples from 5–6 mice, *p<0.05, **p<0.01, *** p<0.001. For multiple comparisons, data was analysed by two-way ANOVA using Tukey’s multiple comparison test.

**Fig 7:**
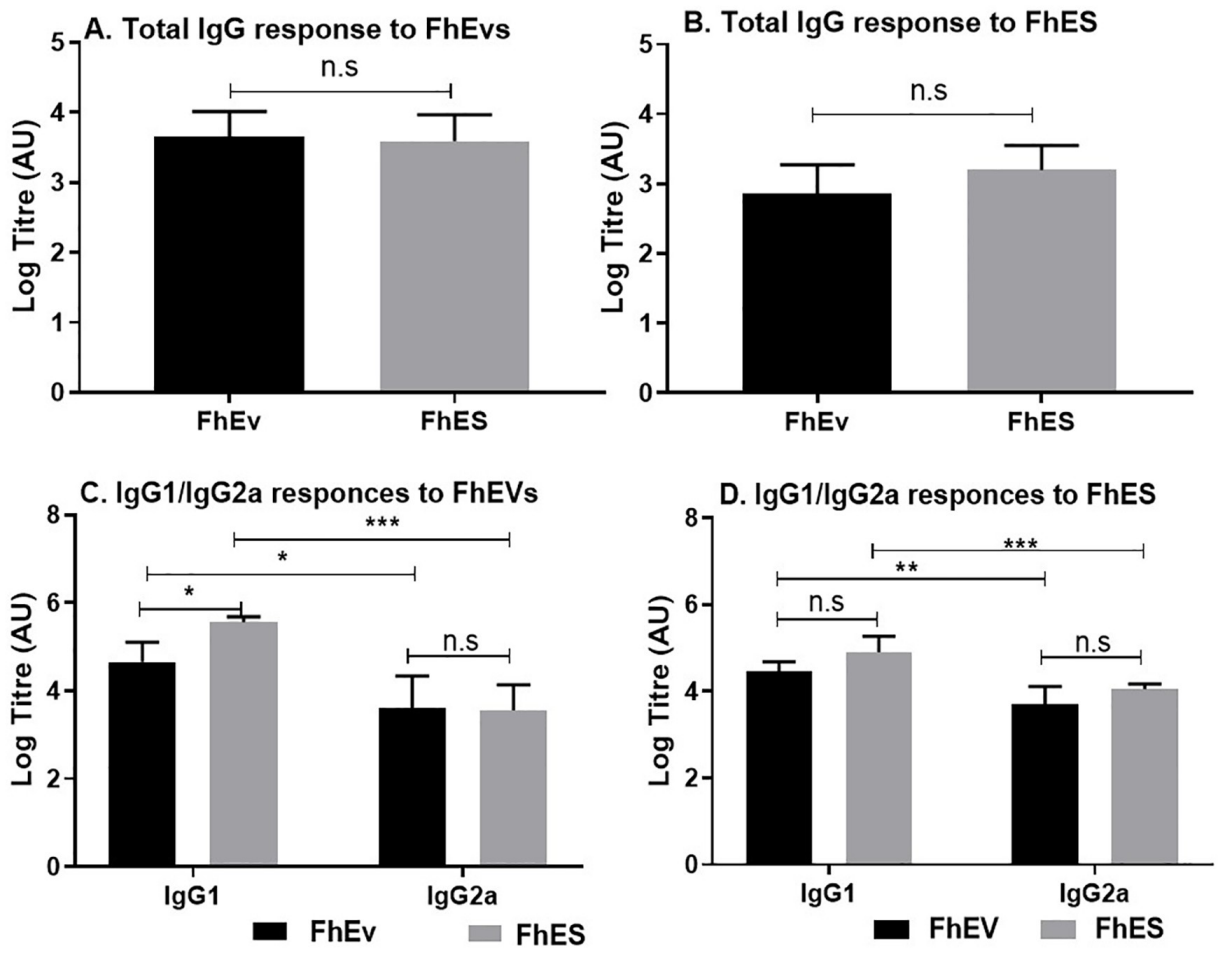
Mice immunised with FhEVs formulated in Alum induce strong antigen specific antibody responses. 7–8 Mice were injected with Alum, FhEVs (in alum) or FhES (in alum) on day 0, 14 and 28. After 2 weeks’ serum was isolated to measure Total IgG, IgG1 and IgG2a specific FhEV and FhES antibody responses. Data shown is presented as the mean ± SD of three replicate samples from 7–8 mice, *p<0.05, **p<0.01, *** p<0.001. For multiple comparisons, data was analysed by two-way ANOVA using Tukey’s multiple comparison test (C). Otherwise, student t-test was used for differences between means of control and treated (A, B).

To determine if FhEVs can induce adaptive immune responses *in vivo*, mice were immunised with purified total FhEVs alone and in a formulation with alum. No antigen-specific IL-4, IFNγ, IL-13 or IL-10 immune responses were detected in splenocytes obtained from mice immunised with FhEV or PBS alone (S2A–S2D Fig), although low but significant levels of FhEV-specific total IgG antibodies (p<0.05) was detected (Fig 5C) indicating the induction of T-cell independent antibody responses. By contrast, statistically significant levels of antigen-specific IFNγ (p<0.01) and IL-2 (FhEV p<0.01; FhES p<0.001) was observed when either FhEV, and FhES, were formulated in alum. IL-5 (p<0.05) was also detected in FhES immunised mice (Fig 6). No antigen specific induction of IL-10 or IL-17 was detected in all groups examined (S1E and S1F Fig). A significant increase in total IgG, IgG1, and Ig2a antibodies (p<0.05) were detected in the sera of mice immunised with alum-formulated FhEV and FhES (Fig 7).

## Discussion

The release of extracellular vesicles (EVs) by parasitic helminths is a mechanism by which these pathogens deliver a variety of molecules to host cells and tissues. Understanding the composition of EVs of different helminth parasites is important not only to learn how they are utilised in host-pathogen communication but also because they are a rich source of molecules for vaccine development and for the discovery of diagnostics and biomarkers of infection. This study investigated a gravity flow procedure developed by Muscante *et al*. [37] to isolate exosomes from clinical urine samples. We considered that this method would be very suitable for isolating *F*. *hepatica* EVs (FhEVs) from ES products, which are similarly highly diluted samples. We show that large volume of ES products can be reduced within a dialysis tubing (MWCO 1000kDa) to a small volume that retains a spectrum of intact *F*. *hepatica*-released EVs. TEM showed that the FhEVs consisted of a wide variety of sizes ranging from 30 to 200 nm demonstrating the breath of vesicles released by the parasite. The major GO terms associated with proteins identified by proteomic analysis included vesicle mediated transport, vesicle fusion, extracellular, exosome, exocytosis, endocytosis, and ESCRT 1 complex, endorsing the method for FhEVs isolation.

We compared the proteomic composition of FhEVs isolated by gravity flow to two populations of EVs isolated by sequential centrifugation, which we termed 15K- and 120K EVs [34]. We expected that the forces exerted by centrifugation and the resuspension of EV pellets would be more harsh and could potentially damage the vesicles. However, we found that 72% of the 618 proteins identified in the gravity-flow EVs were identical to proteins isolated in both 15K and 120K EVs. Proteins exclusive to the gravity flow EVs only represented 11% of the total protein contents and 14 of the 25 most abundant proteins were also common to all three EVs types (most predominantly, thioredoxin, leucine aminopeptidase, HSP-70, annexin, and hexokinase). Furthermore, 183 surface-exposed proteins, most with N- and/or *O*-glycosylation sites, were shared between the EVs. Comparative proteomic analysis of the FhEVs with the ES depleted of EVs by centrifugation identified 159 proteins including structural proteins, metabolic enzymes, and key proteins typically associated with the adult *F*. *hepatica* secretome, such as peptidases, peptidase inhibitors, and anti-oxidant proteins. Several of these proteins are encoded by multi-copy gene families. Representatives of these families were identified within both the EV samples and the EV-depleted ES samples, which may be as a result of the methodology used to recover the EVs and/or release following damage to the EVs. This result may also imply that the proteins within these families have different functions and are released by the parasite into the environment via numerous routes as free proteins as well as being contained within the EVs. Lastly, lectin microarray analysis of the three sets of EVs revealed a similar surface glycan topology with a predominance of high mannose glycoproteins. Collectively, our data suggests that EVs isolated by gravity flow consisted of both 15K and 120K populations of EVs and that EVs isolated by centrifugation are not necessarily damaged by the process.

Structural proteins such as universal stress proteins (USPs), annexins, and syntenin-1 are among the most abundant proteins identified in the *F*. *hepatica* EVs. USPs have been previously described as highly expressed in helminths such as Fasciola and Schistosoma and are critical to enabling the parasite to withstand unfavourable environmental conditions such as oxidative stress, temperature fluctuations, low pH, and/or hypoxia during its complex developmental lifecycle [51]. Homogenous USPs are not expressed in human, ovine and bovine since these gene families were lost during evolution [52] and thus they could represent good candidates for selectively targeting the parasite via vaccines or chemotherapeutics. On the other hands, annexins are ubiquitous soluble proteins involved in critical cellular processes such as anti-inflammatory, membrane repair, cell migration, cell proliferation, and apoptosis [53]. They have been described previously in helminths including Fasciola [34], Schistosoma [54], Clonorchis, [55] and Taenid Cestodes [56] species, and typically found localised to the surface tegument and apical membrane. A recent study reported that five of the 13 Schistosoma annexins identified were conserved among *S*. *mansoni*, *S*. *japonicum*, and *S*. *haematobium* species [54]. The scaffold protein syntenin was localised to the gut epithelia of Schistosoma during the intravascular stages of its lifecycle. Mice vaccinated with recombinant Schistosoma syntenin with Freund’s adjuvant elicited a Th1 immune response that was partially protective [57].

Several studies have examined the potential of vesicle trafficking molecules as a mean of controlling disease pathology including studies on parasite infection [25,58]. These targets include VPS36, a subunit of ESCRT machinery important for multi-vesicular biogenesis and exocyst complex components (ECC-1, -2, and -8) involved in docking exocytic vesicles with fusion sites on the plasma membrane [25,58]. Other interesting targets are flotillins because they are associated with membrane rafts implicated in growth factor signalling, endocytosis, cell migration, cell differentiation, and membrane trafficking. Flotillin 1 is expressed at the apex of *Eimeria tenella* sporozoites, a region mediating host cell invasion. Pre-treatment of *E*. *tenella* sporozoites with flotillin-1 blocking antibodies inhibited parasite invasion of host cells [59]. Flotillins are also important in maintaining and regenerating the central nervous system through regulation of neoblast cell proliferation in the planarian species, *Dugesia japonica* [60], however, its role in *F*. *hepatica* biology has yet to be examined.

The immune modulatory properties of isolated FhEVs revealed that they induced a DC population that differed to DCs characterised previously from *F*. *hepatica*-infected animals [61]. FhEVs-stimulated DCs secreted significant levels of TNF-α with enhanced expression of co-stimulatory markers (CD80, CD86, CD40, OX40L), SIGNR1, and intracellular signalling molecules (SOCS1 and SOCS3). Typically, helminth infection or their major secretory molecules supress these co-stimulatory markers [36,49,61]. In contrast, FhEVs induced a semi-mature DC phenotype that was confirmed by the lack of ICAM-1 expression, an extracellular adhesion molecule that correlates with antigen-presentation properties and a hallmark of DC activation [62,63]. When co-cultured with CD4^+^ cells, FhEVs inhibited antigen-specific IL-2 production, with no influence on all other cytokines measured except a reduction in IFNγ in polyclonal-activated CD4^+^ cells. Whether, the lack of IL-2 augments or inhibits immune function has not been determined [64]. However, FhEVs activated DCs do not induce IL-13 or IL-10 when co-cultured with CD4^+^ cells and therefore unlike other major components of the secretome do not have an important role in activating Th2 or Tregs CD4^+^ cells that are typically associated with *F*. *hepatica* infection [65].

FhEVs enhanced OX40L on DCs, a molecule that interacts with OX40 on CD4^+^ T-cells amplifying Th2-cell polarisation [66]. OX40L is highly expressed on innate immune cells such as ILC2 cells, macrophages, B-cells, and a subtype of DCs (DC2) from hosts with helminth infection or allergy [66]. Nevertheless, FhEV-activated DCs did not enhance Th2 cytokine production when co-cultured with CD4^+^ cells suggesting that, at least in this instance, OX40L may not be physiologically relevant to the modulatory effects exhibited by *F*. *hepatica* to induce Th2-cellular responses. Similarly, studies in helminth and allergy have shown increased expression of SIGNR1 on innate immune cells enhanced the recognition and processing of molecules that send signals enhancing Th2 immune responses [67,68]. In this study, SIGNR1 expressed by FhEV-stimulated DCs, however DCs stimulated with FhEV did not activate Th2 immune responses.

FhEVs enhanced SOCS protein expression in DCs, negative regulators of cytokine signalling that act as a negative feedback mechanism to dampen immune responses. In contrast to its suppressive effects detailed in the literature [69,70], the induction of SOCS molecules by FhEVs does not influence the subsequent inflammatory signalling as FhEV-activated DCs respond to LPS. This contrasts with previous studies that showed hypo-responsive state in DCS stimulated with LPS either during *F*. *hepatica* infection or following stimulation with parasite antigens [36,49]. Given the powerful immune modulatory properties exhibited by the parasite during infection, perhaps EV-packaged antigens are not as critical as other immune modulators released by the parasite secretome [35,36,49].

A recent study of *Echinostoma caproni*-infected mice showed delayed parasite development and reduced severity of infection following immunization with parasite exosomes [71] providing a promising outlook for the future use of EVs as vaccines or vaccine delivery systems. However, mice vaccinated with FhEVs induced T-cell independent antibody responses. The inhibitory effect of FhEVs on IL-2 production could offer a reasonable explanation, as IL-2 is critical for the initiation and propagation of robust T-cell responses that in turn induce robust antibody production from B-cells [72]. This study suggests that FhEVs were tolerated and, in contrast to the study by Trelis *et al*. [71], FhEVs do not display adjuvant-like properties.

FhEVs immunised with adjuvant (Alum), induced a mixed Th1/Th2-cell response with significantly more cytokine observed when CD4^+^ cells were stimulated with FhES compared to FhEVs. Immunisation with FhEVs induced strong total IgG, IgG1, and IgG2a antibody titres supporting the mixed Th1/Th2 response with no overall significant differences in antibody titres observed. However, while these studies demonstrated that FhEVs could induce a good immune response showing promise as a vaccine candidate, the vaccination delivery system must be optimise to induce strong Th1 immune responses required for protection [73]. Rats vaccinated with total adult homogenate with either CPG or Freunds complete adjuvant (FCA) also induced a mixed Th1/Th2 immune response demonstrating that these adjuvants may not be optimal [74]. Particularly, since FCA is no longer licenced for use in ruminants [75] and initial studies using more TLR-based adjuvants in livestock did not result in enhanced protection [76,77]. Many recently developed Fasciola-based vaccines use molecules that suppress TLR ligand activation, which limits the use of TLR-based adjuvants as a component of any *F*. *hepatica* vaccine formulation [78]. This may explain the limited success of these adjuvants when added to vaccine formulations [32]. However, in the context of exosome-based vaccines, TLR-based adjuvants maybe useful given that FhEV can modulate DCs independent of TLR2 and TLR4. There are a new generation of veterinary adjuvants that were recently examined in experimental models. Adjuplex, a biodegradable matrix derived from soy lecithin, is potent and well tolerated as a veterinary vaccine. When administered with myosin regulatory light chain it induced strong protective Th1 immune responses in rats challenged with *F*. *hepatica* infection [79]. Similarly, naltrexone, an opioid receptor antagonist that can shift the immune response toward a Th1 profile induced strong protective Th1 immune responses in mice when administered with alum and *F*. *hepatica* ES antigens [80]. Finally, in human vaccine development a new generation of TLR-based adjuvants licensed for use such as MPL (3-O-desacyl-4-monophosohryl lipid A), a detoxified bacterial lipopolysaccharide in aluminium salts, could be a promising source of novel adjuvants in veterinary vaccines [81].

This study adds to the growing immuno-proteomic database that will be an important source for the discovery of future parasite vaccines and immunotherapeutic biologicals. It also shed light on the biology of *F*. *hepatica* vesicles. There are many immune modulatory molecules packaged within the FhEVs of adult *F*. *hepatica* parasites [34] that are phagocytosed by DCs and other antigen presenting cells during infection. However, FhEVs exhibit properties that are very different to that observed for individual molecules or for immune cells stimulated with *F*. *hepatica* ES products. Therefore, it is clear that the response during infection cannot be attributed to one molecule alone but rather a range of molecules that send multiple signals through many receptors activating different intracellular pathways that ultimately lead to the strong Th2 and suppressive responses observed with *F*. *hepatica* infection. While in the last few years there are significant advances in the field of EV research, further work is required in order to understand the important role FhEVs play in parasite biology and to further examine their potential as therapeutic target.

## Supporting information

S1 Fig BMDCs were incubated with PBS, FhEVs, LPS or FhEV/LPS for 18 hours and supernatant was removed to measure (A) IL-10 (B) IL-12p70 and (C) TNF-α by commercial ELISA. BMDCs were incubated with FhEVs in the presence of PBS, anti-TLR2, and anti-TLR4 blocking antibody for 18 hours and supernatant was removed to measure (D) TNF-α by commercial. Cell surface expression of **(E)** ICAM, **(F)** MR, and **(D)** Dectin on FhEVs stimulated dendritic cells after 18 hours. Data shown is a representative of three independent experiments presented as the mean ± SD of three replicate samples, *p<0.05, **p<0.01, *** p<0.0001. Student t-test was used for differences between means of control and treated and between fold change between control and treated. BMDCs treated with OVA peptide were also adoptively transferred over the sternum of OT-II mice. After 7 days, sdLN were removed for re-stimulation with OVA peptide or PMA (20ng/ml) and anti-CD3 (1μg/ml) for 72 hours and **(H)** IL-13 and **(I)** IL-10 measured by commercial ELISA. Data shown is the mean ± SD of three replicate samples from 8 mice, *p<0.05, **p<0.01, *** p<0.001. For multiple comparisons, data was analysed by two-way ANOVA using Tukey’s multiple comparison test.
(TIF)

S2 Fig 5–6 mice were injected with PBS or FhEVs on day 0 and 14. After 2 weeks’ spleens were removed for re-stimulation with PBS, FhEVs, FhES or PMA (20ng/ml) and anti-CD3 (1μg/ml) for 72 hours and IFN-γ (A), IL-2 (B), IL-10 (C) and IL-13 (D) measured by commercial ELISA. Data shown is presented as the mean ± SD of triplicate samples from 5–6 mice, *p<0.05, **p<0.01, *** p<0.001. For multiple comparisons, data was analysed by two-way ANOVA using Tukey’s multiple comparison test. **7**–8 Mice were injected with Alum, FhEVs (in alum) or FhES (in alum) on day 0, 14 and 28. After 2 weeks’ spleens were removed for re-stimulation with PBS, FhEVs, FhES or PMA (20ng/ml) and anti-CD3 (1μg/ml) for 72 hours and IL-17 (E) and IL-10 (F) measured by commercial ELISA. Data shown is presented as the mean ± SD of triplicate samples from 7–8 mice, *p<0.05, **p<0.01, *** p<0.001. For multiple comparisons, data was analysed by two-way ANOVA using Tukey’s multiple comparison test.
(TIF)

S1 Table Identification of proteins by LC-MS/MS within the proteome of F. hepatica total EV population isolated by gravity flow compared with the 15K and 120K specific populations isolated by differential centrifugation. (XLSX)

S2 Table Gene Ontology (GO) analysis of the 618 proteins identified from the FhEVs isolated by gravity flow. (XLSX)

S3 Table Description of proteins putatively associated with the surface of FhEVs and their predicted signal peptide and glycosylation site characteristics. (XLSX)

S4 Table List of plant lectins and their binding specificities used in the glycan microarray profiling of FhEVs and FhES. (XLS)
