# Supplementary figures and images for: Fasciola hepatica Extracellular Vesicles isolated from excretory-secretory products using a gravity flow method modulate dendritic cell phenotype and activity
Source: PLoS Negl Trop Dis. 2020 Sep 8;14(9):e0008626. doi: 10.1371/journal.pntd.0008626 (PMC7521716; doi:10.1371/journal.pntd.0008626)

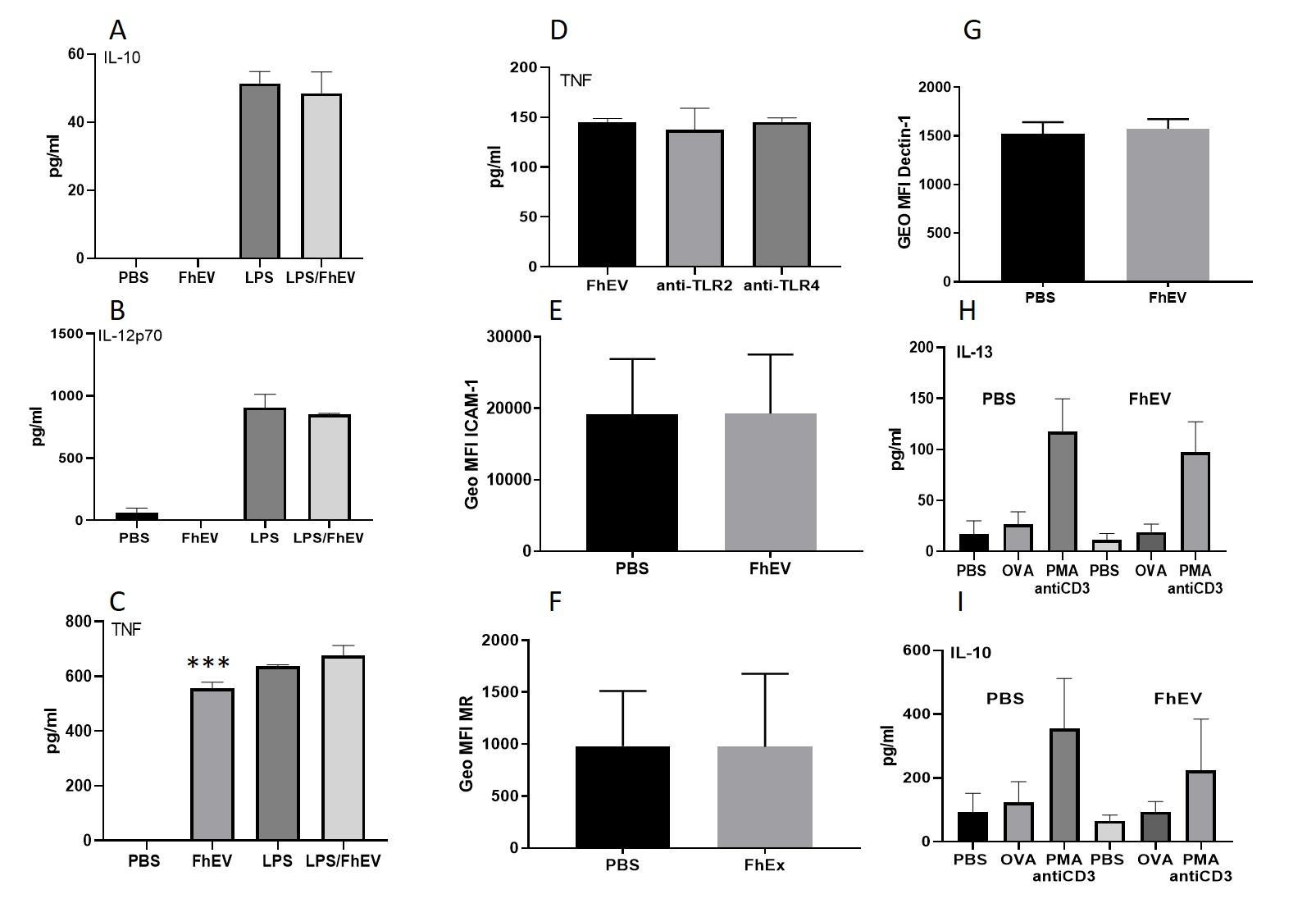

Supplement: S1 Fig — BMDCs were incubated with PBS, FhEVs, LPS or FhEV/LPS for 18 hours and supernatant was removed to measure (A) IL-10 (B) IL-12p70 and (C) TNF-α by commercial ELISA. BMDCs were incubated with FhEVs in the presence of PBS, anti-TLR2, and anti-TLR4 blocking antibody for 18 hours and supernatant was removed to measure (D) TNF-α by commercial. Cell surface expression of (E) ICAM, (F) MR, and (D) Dectin on FhEVs stimulated dendritic cells after 18 hours. Data shown is a representative of three independent experiments presented as the mean ± SD of three replicate samples, *p<0.05, **p<0.01, *** p<0.0001. Student t-test was used for differences between means of control and treated and between fold change between control and treated. BMDCs treated with OVA peptide were also adoptively transferred over the sternum of OT-II mice. After 7 days, sdLN were removed for re-stimulation with OVA peptide or PMA (20ng/ml) and anti-CD3 (1μg/ml) for 72 hours and (H) IL-13 and (I) IL-10 measured by commercial ELISA. Data shown is the mean ± SD of three replicate samples from 8 mice, *p<0.05, **p<0.01, *** p<0.001. For multiple comparisons, data was analysed by two-way ANOVA using Tukey’s multiple comparison test. (TIF) [file pntd.0008626.s001.tif]

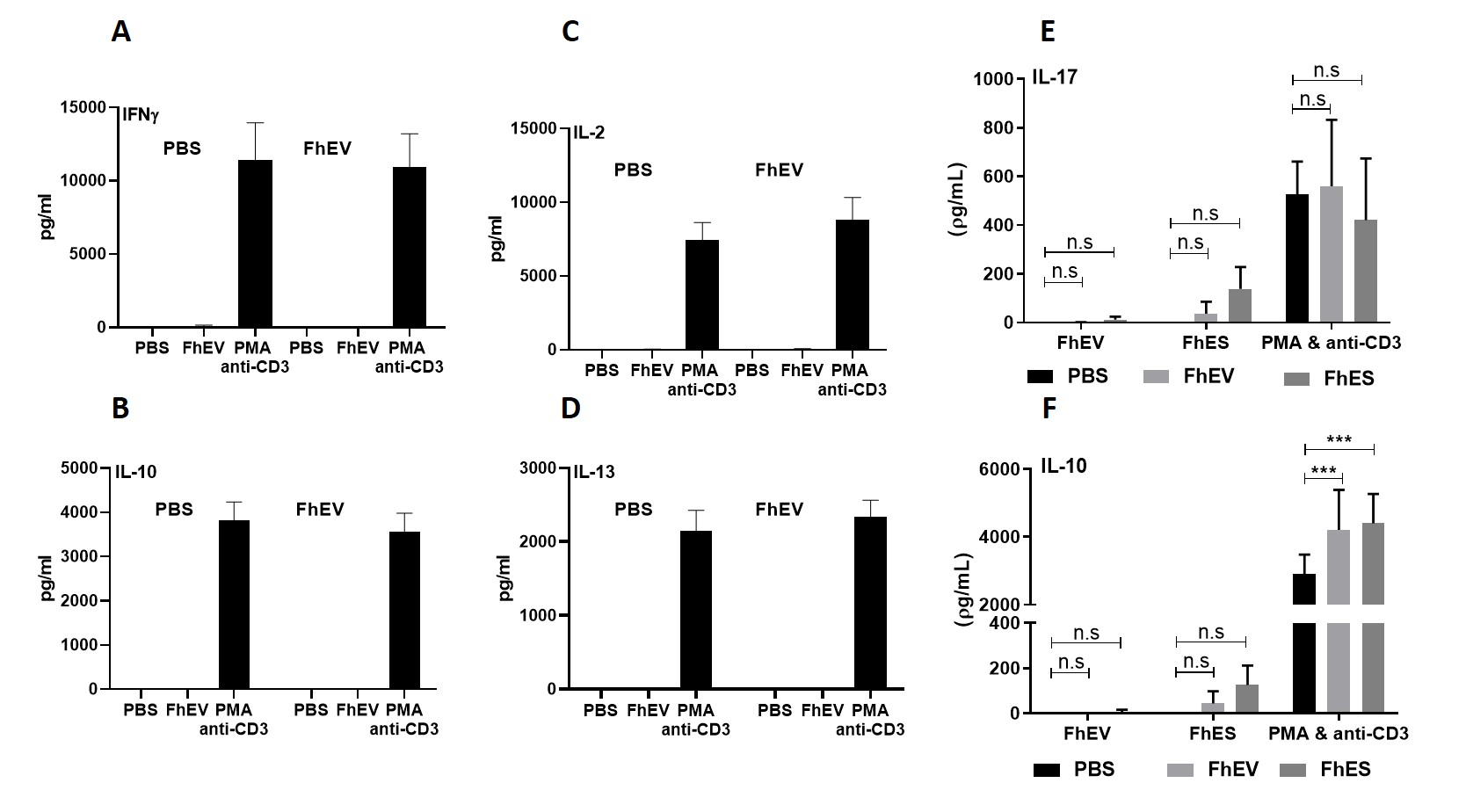

Supplement: S2 Fig — After 2 weeks’ spleens were removed for re-stimulation with PBS, FhEVs, FhES or PMA (20ng/ml) and anti-CD3 (1μg/ml) for 72 hours and IFN-γ (A), IL-2 (B), IL-10 (C) and IL-13 (D) measured by commercial ELISA. Data shown is presented as the mean ± SD of triplicate samples from 5–6 mice, *p<0.05, **p<0.01, *** p<0.001. For multiple comparisons, data was analysed by two-way ANOVA using Tukey’s multiple comparison test. 7–8 Mice were injected with Alum, FhEVs (in alum) or FhES (in alum) on day 0, 14 and 28. After 2 weeks’ spleens were removed for re-stimulation with PBS, FhEVs, FhES or PMA (20ng/ml) and anti-CD3 (1μg/ml) for 72 hours and IL-17 (E) and IL-10 (F) measured by commercial ELISA. Data shown is presented as the mean ± SD of triplicate samples from 7–8 mice, *p<0.05, **p<0.01, *** p<0.001. For multiple comparisons, data was analysed by two-way ANOVA using Tukey’s multiple comparison test. (TIF) [file pntd.0008626.s002.tif]
